# *Rosmarinus officinalis* L. essential oil enhances salt stress tolerance of durum wheat seedlings through ROS detoxification and stimulation of antioxidant defense

**DOI:** 10.1007/s00709-024-01965-8

**Published:** 2024-06-28

**Authors:** Rania Ben Saad, Walid Ben Romdhane, Alina Wiszniewska, Narjes Baazaoui, Mohamed Taieb Bouteraa, Yosra Chouaibi, Mohammad Y. Alfaifi, Miroslava Kačániová, Natália Čmiková, Anis Ben Hsouna, Stefania Garzoli

**Affiliations:** 1 Centre of Biotechnology of Sfax, Biotechnology and Plant Improvement Laboratory, University of Sfax, B.P ‘1177’, 3018 Sfax, Tunisia; 2 Plant Production Department, College of Food and Agriculture Sciences, King Saud University, P.O. Box 2460, 11451 Riyadh, Saudi Arabia; 3 Department of Botany, Physiology and Plant Protection, Faculty of Biotechnology and Horticulture, University of Agriculture in Kraków, Al. Mickiewicza 21, 31-120, Cracow, Poland; 4 Biology Department, College of Sciences and Arts Muhayil Assir, King Khalid University, 61421 Abha, Saudi Arabia; 5 Faculty of Sciences of Bizerte UR13ES47, University of Carthage, BP W, 7021 Jarzouna, Bizerte, Tunisia; 6 Biology Department, Faculty of Science, King Khalid University, 61421 Abha, Saudi Arabia; 7 Faculty of Horticulture, Institute of Horticulture, Slovak University of Agriculture, Tr. A. Hlinku 2, 949 76 Nitra, Slovakia; 8 School of Medical & Health Sciences, University of Economics and Human Sciences in Warsaw, Okopowa 59, 01-043 Warsaw, Poland; 9 Department of Environmental Sciences and Nutrition, Higher Institute of Applied Sciences and Technology of Mahdia, University of Monastir, 5100 Mahdia, Tunisia; 10 Department of Chemistry and Technologies of Drug, Sapienza University, P.le Aldo Moro 5, 00185 Rome, Italy

**Keywords:** Durum wheat, Rosemary essential oil, Biostimulant, Antioxidant enzymes, Salt stress

## Abstract

Salt-induced stress poses a significant barrier to agricultural productivity by impeding crop growth. Presently, environmentalists are dedicated to safeguarding food security by enhancing agricultural yields in challenging environments. Biostimulants play a crucial role in mitigating abiotic stresses in crop production, and among these, plant essential oils (EOs) stand out as organic substances with diverse biological effects on living organisms. Among the natural promoters of plant growth, *Rosmarinus officinalis* L. essential oil (*Ro*EO) has gained considerable attention. Although the manifold effects of essential oils (EOs) on plant growth have been extensively demonstrated, their impact on salt stress tolerance in durum wheat seedlings remains unexplored. This investigation was undertaken to evaluate the biostimulatory capabilities of *Ro*EO on the durum wheat cultivar “Mahmoudi.” The effects of three *Ro*EO concentrations (1, 2.5, and 5 ppm) on seed germination, growth establishment, and the induction of salt resistance under salinity conditions (150 mM NaCl) were tested. At 5 ppm, *Ro*EO enhanced seedlings’ tolerance to salinity by improving growth and reducing membrane deterioration and oxidative stress-induced damage. The expression profile analyses of seven stress-related genes (*TdNHX1*, *TdSOS1*, *TdSOD*, *TdCAT*, *TdGA20-ox1*, *TdNRT2.1*, and *TdGS*) using RT-qPCR showed enhancement of several important genes in durum wheat seedlings treated with 5 ppm *Ro*EO, even under control conditions, which may be related to salt stress tolerance. The results indicate that the application of *Ro*EO suggests a possible alternative strategy to increase salt tolerance in durum wheat seedlings towards better growth quality, thus increasing ROS scavenging and activation of antioxidant defense.

**Supplementary information:**

The online version contains supplementary material available at 10.1007/s00709-024-01965-8.

## Introduction

Rising climate change has precipitated a myriad of environmental challenges, leading to a decline in crop productivity. Among these issues, salinity has emerged as a particularly detrimental abiotic stressor that impedes both seed germination and seedling growth (Xu et al. 2017). The impact of salt stress on cereal productivity significantly influences the pillars of sustainable food security and overall food production (Alkharabsheh et al. 2021). Wheat holds paramount importance as a global cereal crop, serving as a vital source of calories and protein essential for the world’s human population. It boasts the largest harvested area and ranks as the second-highest in terms of production rate among cereals. Wheat is subjected to a variety of abiotic stresses in the arid Mediterranean region, including high temperatures, drought, and salinity, which severely affect plant development as well as yield and quality losses (Laraus 2004). In the realm of agriculture, the escalating demand for sustainability underscores the imperative to transition from synthetic fertilizers to natural or biological alternatives (Tauler and Baraza 2015; Tahami et al. 2017). To meet this objective, biostimulants and bio-protectants have been put forth as promising agents with the potential to foster plant growth and enhance overall crop yields (Bulgari et al. 2019; Singh et al. 2020). Organic acids, amino acids, and compounds like humic acid, salicylic acid, and polyamines stand out as stimulants. Their application, whether applied to the soil or foliage, typically enhances various plant growth-related traits (Souri 2016; Canellas et al. 2015; Marschner 2011). There is evidence to suggest that a diverse array of biostimulants has the capacity to enhance the tolerance of plants to adverse conditions such as drought, salinity, and cold stress (Van Osten et al. 2017). Recent studies have suggested that the volatile compounds found in EOs may offer advantages to plants thriving in challenging or unfavorable environments (Hara et al. 2013; Yamauchi et al. 2015). A distinct category of well-defined biostimulants comprises EOs, which are intricate natural products typically extracted from aromatic plants. These oils exhibit a broad spectrum of biological activities, including resistance against diseases and pests, and they contribute to enhancing plant stress tolerance in the face of various abiotic stress factors (Kahramanoglu and Usanmaz 2021; Kesraoui et al. 2022). In natural settings, EOs influence the growth of neighboring plants, particularly those in proximity to essential oil-producing plants. Certain EOs serve as allelochemicals, exerting inhibitory effects on seed germination (Koul et al. 2008). Their facile metabolic processes have opened avenues for their application in agricultural settings, supplanting the use of synthetic chemicals (Belasli et al. 2020). Utilizing EOs in conjunction with seed coating and priming technologies holds significant promise as a natural biostimulant, contributing substantially to the improvement of both biotic and abiotic stress resistance (Tavares et al. 2002; Lutts et al. 2016). Numerous studies have predominantly concentrated on exploring the antifungal, antibacterial, and insecticidal properties of plant EO (Ben Hsouna et al. 2013, 2019, 2022; Belasli et al. 2020; Moumni et al. 2021; Ben Akacha et al. 2022; Kesraoui et al. 2022). Additionally, there is a growing interest in elucidating the role of EOs in conferring abiotic stress tolerance during the critical stages of germination and seedling growth (Bi̇ngöl and Battal 2017; Souri and Bakhtiarizade 2019; Ben-Jabeur et al. 2019). The application of essential oils or plant extracts as a pre-treatment for seeds has been shown to induce a biostimulant effect, enhancing tolerance to drought stress (Farooq et al. 2018; Ben-Jabeur et al. 2022). The methods employed in seed preparation exert a notable influence on the metabolic, biochemical, and enzymatic activities of the seed (Nile et al. 2013). By employing seed priming, early germination and robust seedling development can be achieved, particularly under arid conditions (Raj and Raj 2019; Zulfiqar 2021).

Rosemary (*Rosmarinus officinalis* L.), a perennial herb belonging to the Lamiaceae family, originates from the Mediterranean region. Renowned for its resilience, it has become prevalent in numerous countries, attributed to its essential oils, extracts, use as a spice, and diverse biological effects on plants (Jordán et al. 2013). The EOs derived from this plant exhibit a multitude of pharmacological properties (Lemos et al. 2015). The leaves of this plant are abundant in aromatic compounds, as well as iron, calcium, and vitamin B6, imparting a broad spectrum of medicinal and health-related benefits in human health programs (Jalali-Heravi et al. 2011). Rosemary EO showcases a diverse array of properties, including antioxidant, antimicrobial, anti-inflammatory, and insecticidal activities (Abo Ghanima et al. 2020; Da Silva Bomfim et al. 2020; Hashemi Gahruie et al. 2017).

The primary objective of this study was to investigate the biostimulatory properties of *Ro*EO concerning germination, growth, salt tolerance, and antioxidant systems (SOD, CAT, and GPX) in durum wheat seedlings under salt stress conditions. Furthermore, our goal was to elucidate how *Ro*EO regulates the enhancement of salt stress resistance in durum wheat, with a focus on osmotic regulation, redox homeostasis, and molecular responsiveness during the seedling stage.

## Materials and methods

### Plant materials

The EOs extracted from the needles of *Rosmarinus officinalis* L., cultivated in Tuscany, Italy, and obtained through steam distillation, were supplied directly by “èssenziale” Azienda Agricola, San Donato in Poggio (FI), Italy. The plants used for extraction were collected in June 2022.

### GC–MS analyses of *Ro*EO

The chemical composition of the rosemary essential oil (*Ro*EO) was characterized using a Perkin Elmer Clarus 500 (Waltham, MA, USA) model gas chromatograph coupled to a mass spectrometer equipped with a flame ionization detector (FID) (Ben Akacha et al. 2023). Chromatographic separation of components was achieved using a Stabil wax fused-silica capillary column (Restek, Bellefonte, PA, USA) with dimensions of 60 m × 0.25 mm and a 0.5-mm film thickness. The oven temperature program started at 60 °C and increased to 220 °C for 20 min at a rate of 6 °C/min. Helium served as the carrier gas with a constant flow rate of 1.0 mL/min. Mass spectra were obtained in electron impact (EI) mode at 70 eV in full-scan mode within the range of 35–550 m/z. Compound identification was performed by matching mass spectra with the Wiley 2.2 (Wiley, NY, USA) and NIST 11 (Gaithersburg, MD, USA) databases. Linear retention indices (LRIs) were calculated using a mixture of C_8_–C_25_ n-alkanes and compared with literature values (Linstrom and Mallard 2014). Relative average percentages were determined by normalizing peak areas without an internal standard and factor correction. The analysis was conducted in triplicate.

### Seed treatment, growth conditions, and measurement of plant growth traits

The experiment utilized seeds from the “Mahmoudi” variety of durum wheat (*Triticum turgidum* L. var. durum), a widely cultivated Tunisian variety, which were supplied by the Centre d’Appui Chebika-CRDA Kairouan in Tunisia. The seeds underwent surface sterilization following the method described by Bouteraa et al. (2022). To avoid disruption of the normal physiological processes of plants, stunted growth, wilting of leaves, and even plant death, the *Ro*EO was used in moderation and at appropriate concentrations of 1, 2.5, and 5 ppm with the addition of 0.5% dimethyl sulfoxide (DMSO) as a solubilizing agent to ensure homogenous application of the EO. Subsequently, the seeds were categorized into four groups. The initial group was soaked in distilled water, while the remaining three groups were soaked in 1, 2.5, and 5 ppm *Ro*EO for 12 h. Following the soaking process, the seeds underwent three rinses with distilled water and were subsequently air-dried for 48 h at room temperature. Thereafter, 20 seeds were germinated in triplicate using distinct seed batches. The germination process took place on Petri dishes containing sterile filter paper and a half-strength MS medium supplemented with varying concentrations of *Ro*EO (0, 1, 2.5, and 5 ppm). The Petri dishes were then positioned in a growth chamber at 22 °C ± 2.0 with a 12-h photoperiod and 65% relative humidity. The assessment of germination energy (GE) was conducted on the 3rd day post-sowing and was calculated as follows: GE = (number of germinated seeds on day 3/total number of seeds) × 100.

After 8 days of growth, the seedlings were photographed, and seedling length and fresh weight (FW) were determined. The flag leaf area (cm^2^) was determined using UTHSCA image tool program (http://compdent.uthscsa.edu/dig/itdesc.html)*.* The assays were conducted in triplicate using independent seed lots.

### *Ro*EO effect on salt-stressed wheat seedlings

For the salt stress tolerance experiment, wheat seedlings aged 2 days were transplanted into Petri dishes containing half-strength liquid MS medium. This medium was supplemented with 150 mM NaCl and either 0 ppm or 5 ppm of *Ro*EO. The control medium was NaCl-free. The Petri dishes were arranged in accordance with a completely randomized design, with each dish accommodating 20 seeds and each treatment replicated three times. The seedlings were maintained in a constant environment chamber under a 16-h light/8-h dark regime. Nutrient solutions were refreshed every 2 days. After 2 weeks, the seedlings were harvested for subsequent analyses.

### Leaf chlorophyll fluorescence

Chlorophyll fluorescence (*Fv/Fm*) levels were measured on three visually healthy leaves per treatment after a 30-min period of dark adaptation. The quantification was carried out using a portable photosynthesis system, specifically the Handy-Plant Efficiency Analyzer from Hansatech Instruments, following the protocol outlined by Ben Saad et al. (2012).

### Determination of the stress parameters

Electrolyte leakage (El) and the membrane stability index (MSI) were determined following a previously published method by Ben Romdhane et al. (2017). The contents of malondialdehyde (MDA) and H_2_O_2_ were measured as previously described by Bouteraa et al. (2022). Furthermore, fresh leaves from the durum wheat plants were gathered to investigate the accumulation of superoxide radicals (O_2_^−^) via the nitro blue tetrazolium (NBT) staining following the methodology outlined by Ben Saad et al. (2018).

### Determination of osmolyte content

#### Leaf soluble carbohydrates

The quantification of leaf soluble carbohydrates was conducted through the anthrone method. In this process, 0.2 g of fresh leaf tissue was extracted in 2.5 mL of ice-cold 80% ethanol and heated at 95 °C for 60 min (Ben Hsouna et al. 2020). Following extraction, the solution was filtered, and the alcohol was eliminated through evaporation in a hot water bath. Anthrone reagent was employed to prepare the samples, and their absorbance was measured at 625 nm utilizing a spectrophotometer. The carbohydrate content was determined by referencing a standard glucose curve expressed in µg/g FW (fresh weight).

#### Proline content

Free proline concentration in fresh leaves was determined spectrophotometrically at 520 nm according to the method described by Ben Saad et al. (2018, 2024).

### Activity of antioxidant enzymes

The activities of antioxidant enzymes, namely superoxide dismutase (SOD), peroxidase (POD), and catalase (CAT), were quantified spectrophotometrically using methods described in the literature by Ben Saad et al. (2022).

### RNA extraction and quantitative real-time PCR assay

Frozen leaves were utilized for the extraction of total RNA using the RNeasy Plant Mini Kit (Qiagen), following the manufacturer’s instructions. For RT-qPCR, 5 µg of total RNA per sample was employed to synthesize first-strand cDNA using SuperScriptTM III reverse transcriptase (Invitrogen), oligo (dT18), and random hexamer primers, following the manufacturer’s protocols to examine the transcript accumulation of seven stress-related genes, including genes associated with membrane transporters (*TdNHX1*, *TdSOS1*), antioxidant enzymes (*TdSOD*, *TdCAT*), gibberellin oxidase (*TdGA20-ox1*), and genes involved in nitrogen transport and metabolism (*TdNRT2.1* and *TdGS*); total cDNA from durum wheat was employed in RT-qPCR reactions, following the methods outlined by Ben Romdhane et al. (2022, 2024). Relative expression was determined by the comparative threshold cycle (2^−ΔΔCT^) method (Livak and Schmittgen 2001), using the cell division control protein (AAA-superfamily of ATPases) (*CDC* gene) as the housekeeping gene. Three biological replicates were used to calculate relative expression. Primer sequences are listed in supplementary Table S1.

### Statistical analyses

The XLSTAT statistical software (https://www.xlstat.com/en/ (accessed on 10 November 2023)) was used to perform statistical analyses, including one-way analysis of variance and principal component analysis. According to the Bonferroni post hoc test, the means marked with different letters in the graphs differ significantly at *p* < 0.05.

## Results

### Characterization of the chemical volatile composition in *Ro*EO by GC–MS analysis

Twenty compounds in total were detected and identified by GC/MS in *Ro*EO (Table 1). The most abundant compounds, among other substances detected with almost overlapping relative abundances, were camphor (14.4%), 1,8-cineole (13.0%), levoverbenone (11.4%), and *α*-pinene (10.1%). Also significant was the content of the sesquiterpene *β*-caryophyllene (8.6%), as well as that of the three monoterpenes: bornyl acetate (7.9%), limonene (6.4%), and *β*-myrcene (5.2%).

**Table 1:** Chemical volatile composition (percentage mean values ± SD) of *R. officinalis* essential oil (*Ro*EO)

*N*°	Component^1^	LRI^2^	LRI^3^	*Ro*EO^4^ (%)
1	*α*-Pinene	1020	1024	10.1 ± 0.02
2	*β*-Thujene	1115	1117	2.5 ± 0.02
3	*β*-Myrcene	1160	1166	5.2 ± 0.00
4	Limonene	1200	1204	6.4 ± 0.00
5	1,8-Cineole	1208	1206	13.0 ± 0.02
6	3-Heptanone, 6-methyl-	1266	1263	6.6 ± 0.00
7	*p*-Cymene	1285	1282	1.0 ± 0.02
8	3-Octanol	1411	1406	0.2 ± 0.07
9	Filifolone	1426	1423	3.9 ± 0.05
10	1-Octen-3-ol	1457	1453	0.4 ± 0.02
11	Linalool	1508	1500	3.5 ± 0.02
12	Camphor	1533	1528	14.4 ± 0.05
13	Isocamphopinone	1535	1530	1.9 ± 0.08
14	Bornyl acetate	1585	1580	7.9 ± 0.02
15	*β*-Caryophyllene	1597	1594	8.6 ± 0.02
16	L-Pinocarveol	1648	1651	0.2 ± 0.14
17	*α*-Terpineol	1705	1700	1.6 ± 0.02
18	Borneol	1708	1705	0.9 ± 0.02
19	Levoverbenone	1227	1723	11.4 ± 0.07
20	Myrtenol	1808	1804	0.3 ± 0.03
	SUM			100.0
	Monoterpenes			28.7
	Oxygenated monoterpenes			55.5
	Sesquiterpenes			8.6
	Others			7.2

^1^The components are reported according to their elution order on the polar column

^2^Linear Retention Indices calculated using the polar column

^3^Linear Retention Indices from literature

^4^Percentage mean values of *R. officinalis* EO components

### The effects of *Ro*EO on seed germination and growth development of wheat seedlings

**Fig. 1:**
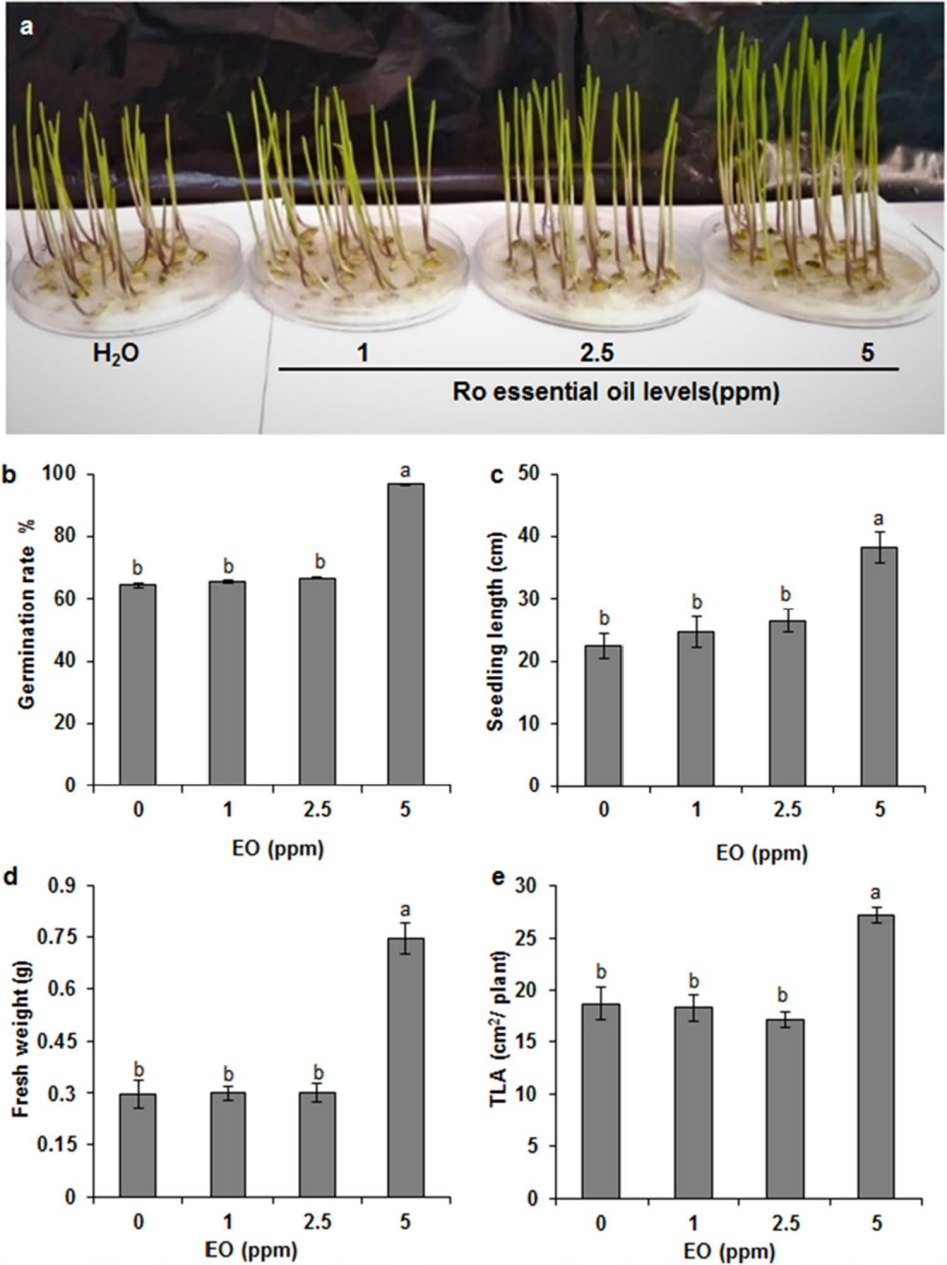
Effect of *Ro*EO at different concentrations (1, 2.5, and 5 ppm) on the seed germination and durum wheat seedlings phenotype. **a** This photo was taken eight days after sowing seeds treated with different *Ro*EO essential oil concentrations. Effect of increasing *Ro*EO concentrations on **b** germination rate, **c** seedling length, **d** fresh weight, and **e** total leaf area (TLA) of durum wheat seeds. Values are the means ± SE of three biological replicates. Means sharing the same letter do not significantly differ at *p* < 0.05. Twenty seeds were assessed for each treatment replicate

Three *Ro*EO concentrations (1, 2.5, and 5 ppm) were applied to determine the effect of the EO on durum wheat germination (Fig. 1). The results showed that seed coating with 5 ppm *Ro*EO significantly increased the germination rate (100%) compared to the control seeds (without *Ro*EO) (64%) (Fig. 1b). At concentrations of 1 and 2.5 ppm of *Ro*EO, seed wheat germinated 2 days earlier than the control seeds; however, the germination rate was unaffected (Fig. 1b). At 5 ppm, *Ro*EO caused seedling elongation (~ 1.7-fold) (Fig. 1c) and increased its fresh weight (~ 2.55-fold) (Fig. 1d), as well as total leaf area (~ 1.5-fold) (Fig. 1e). At this *Ro*EO concentration, the development of the second leaf was more pronounced, while the stem and roots grew longer (Fig. 1) than those of the control and those of the seeds treated with 1 and 2.5 ppm *Ro*EO. As no phytotoxicity symptoms occurred, we considered *Ro*EO at 5 ppm an optimal concentration for wheat seed germination and applied it in subsequent experiments under saline conditions.

### Effects of *Ro*EO on wheat seedling growth, chlorophyll fluorescence, and osmolyte accumulation under salinity

**Fig. 2:**
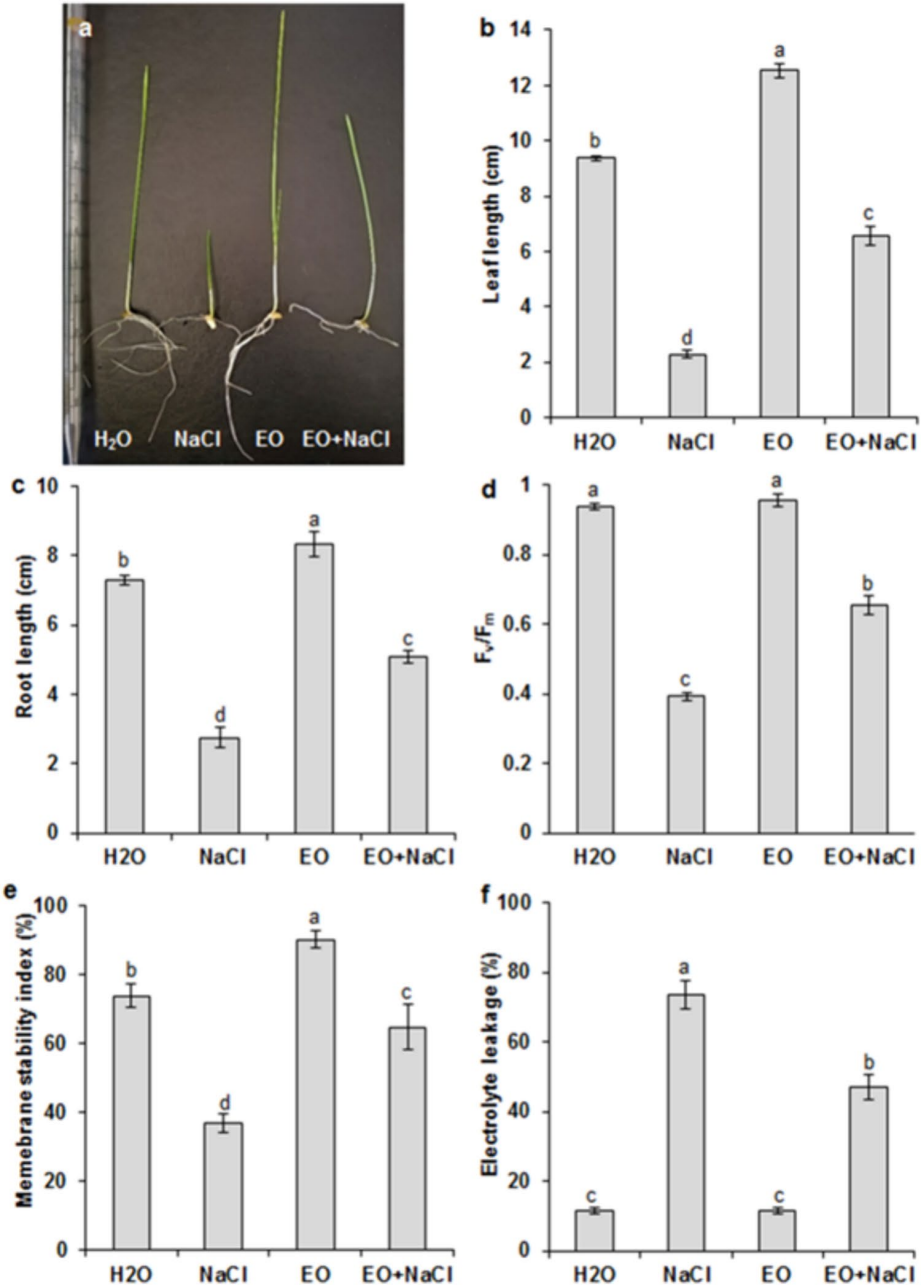
Effect of *Ro*EO at 5 ppm on plant phenotype (**a**), leaf length (**b**), root length (**c**), leaf chlorophyll fluorescence (*Fv/Fm*) (**d**), leaf membrane stability index (MSI) (**e**), and electrolyte leakage (**f**) grown under saline (150 mM NaCl) and non-saline conditions (H_2_O). Values are the means ± SE (*n* = 3). Means denoted by the same letter did not differ significantly at *p* < 0.05

Under salinity (150 mM NaCl), both shoots and roots grew shorter than those of untreated control wheat plants (Fig. 2a). The application of 5 ppm *Ro*EO had a stimulatory effect on wheat seedling growth. When added to salt-treated seedlings, *Ro*EO intensified plant elongation and biomass accretion (Fig. 2a, c). Similarly, total chlorophyll fluorescence (*Fv/Fm*) in the leaves of durum wheat seedlings exposed to salt stress increased by half after the application of 5 ppm *Ro*EO (Fig. 2d). Additionally, *Ro*EO treatment had no effect on the *Fv/Fm* ratio in non-stressed plants. This could be due to the biostimulatory effect of *Ro*EO on the photosynthetic capacity of photosystem II following exposure to salt stress.

### *Ro*EO effect on stress indicators under salinity

**Fig. 3:**
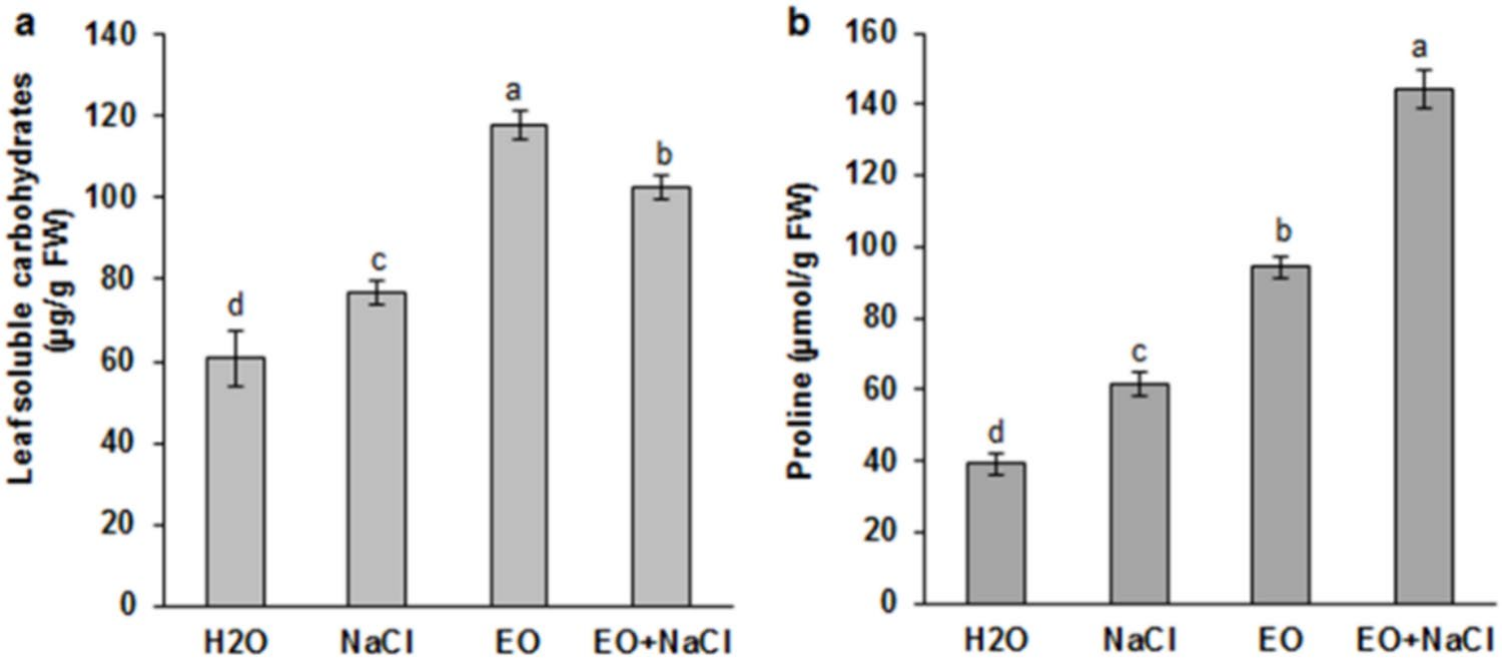
Influence of *Ro*EO application on the leaf soluble carbohydrate content (**a**) and proline content (**b**) of durum wheat seedlings subjected or not to 150 mM NaCl. Values are the means ± SE (*n* = 3). Different letters indicate significant differences at *p* < 0.05

**Fig. 4:**
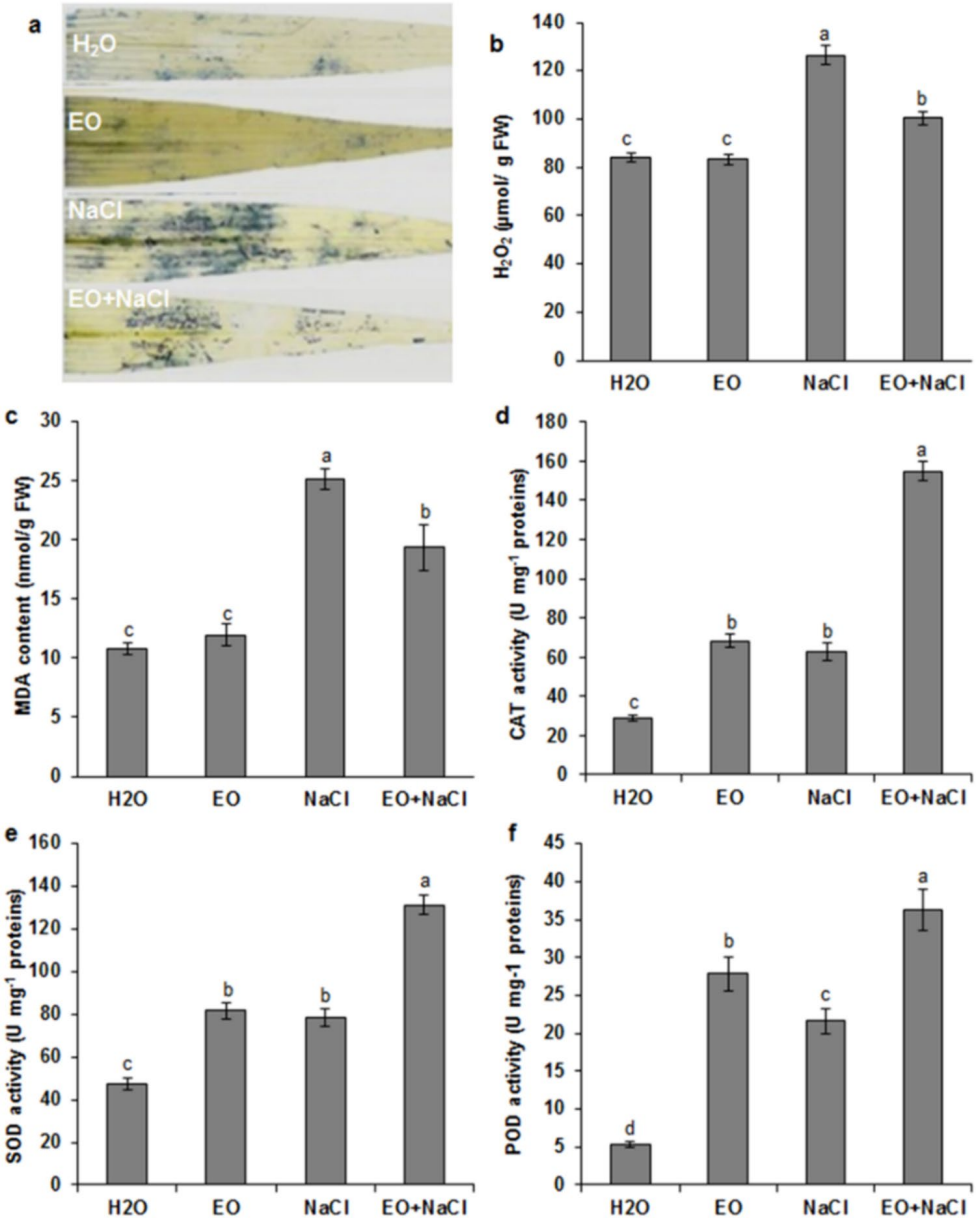
Influence of Ro essential oil application on the contents of H_2_O_2_ (**a**), MDA (**b**), and the activities of SOD (**c**), CAT (**d**), and POD (**e**) in durum wheat seedlings subjected or not to 150 mM NaCl. Values are means ± SEM (*n* = 3). Means sharing the same letter do not significantly differ at *p* < 0.05

*Ro*EO treatment considerably alleviated salinity stress-induced growth reduction, increased MSI, and maintained low EL compared to the plants grown under salt stress conditions (Fig. 2e, f). The influence of *Ro*EO on salt-treated wheat seedlings was monitored via assessment of osmolyte accumulation. The salt-stressed wheat seedlings had significantly higher leaf soluble carbohydrates and proline contents than unstressed seedlings (Fig. 3a, b). *Ro*EO application further increased osmolyte accumulation by ~ 1.5-fold and 1.33-fold, in the case of proline and carbohydrates, respectively (Fig. 3a, b). For the non-stressed plants, *Ro*EO stimulated the accumulation of soluble carbohydrate and proline, by 40% and 71%, respectively, compared to the control conditions. Additionally, the effect of *Ro*EO on oxidative status of wheat seedlings was examined by measuring the changes in the accumulation of the reactive oxygen species H_2_O_2_ and O_2_^−^, as well as in the content of MDA, a marker of lipid peroxidation. As shown in Fig. 4a and c, salt exposure caused a significant increase in foliar H_2_O_2_ and MDA levels (~ 1.56-fold and 2.27-fold, respectively) compared to the control plants. Remarkably, MDA, O_2_^−^, and H_2_O_2_ accumulation was considerably reduced in seedlings treated with *Ro*EO under salt stress conditions (Fig. 4a, c). In the absence of stress, *Ro*EO did not affect H_2_O_2_ and MDA contents.

### The activities of the antioxidant enzymes SOD, CAT, and POD

In comparison with control plants, a marked increase in CAT, SOD, and POD activities was recorded in leaves treated with *Ro*EO alone (by 2-, 1.66-, and 5.2-fold, respectively) and those subjected to salt stress only (Fig. 4d–f). The activities of CAT and SOD were comparable in seedlings treated either with *Ro*EO or salt. The combination of NaCl stress and *Ro*EO treatment tended to enhance CAT, SOD, and POD activities to the greatest extent (~ 5-fold,  ~ 2.8-fold, and ~ 7-fold, respectively, in relation to the control) (Fig. 4d, f).

### Molecular responses of wheat plants treated with *Ro*EO and salinity

**Fig. 5:**
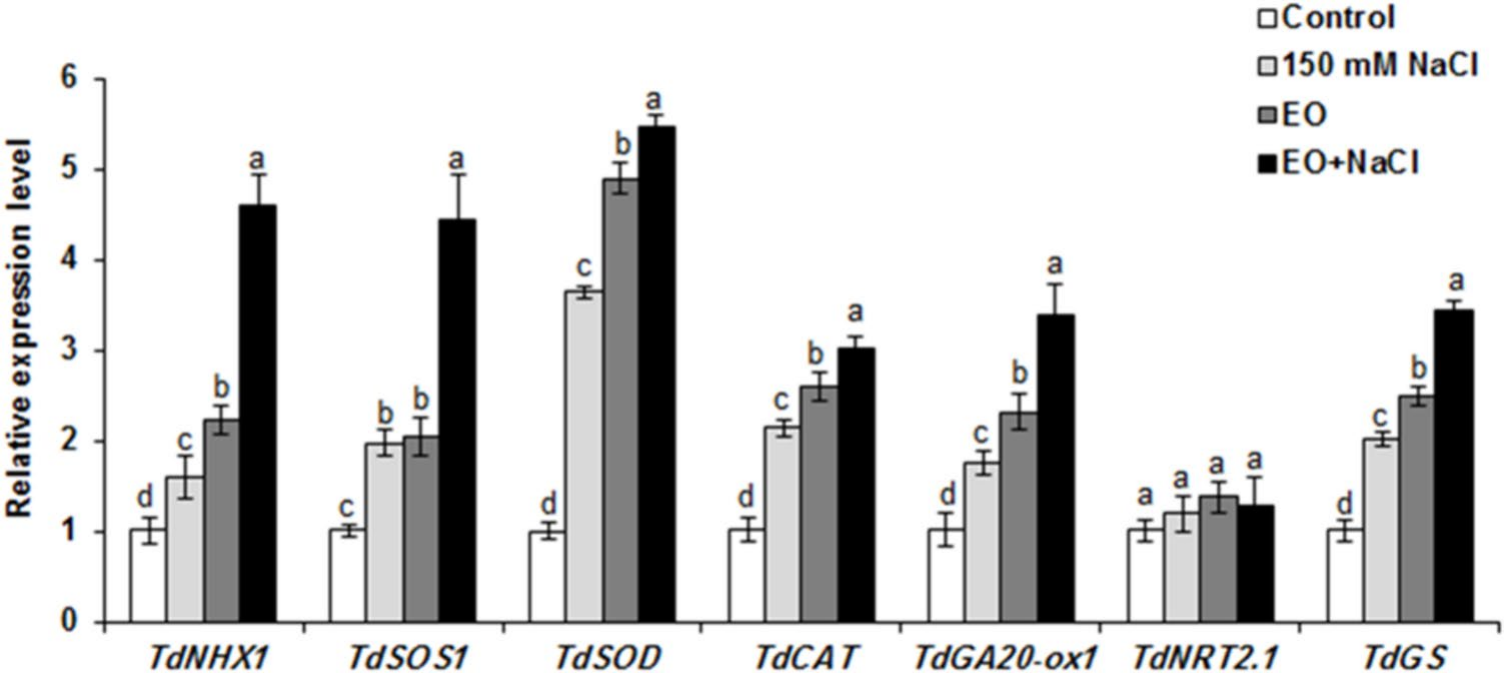
Transcriptional profiles of seven stress-related genes (*TdNHX1*, *TdSOS1*, *TdSOD*, *TdCAT*, *TdGA20-ox1*, *TdNRT2.1*, and *TdGS*) in the leaves of durum wheat seedlings subjected or not to salt stress and 5 ppm *Ro*EO application for 15 days. The data represent means of three independent experiments. Values are the means ± SE (*n* = 3). Means sharing the same letter do not significantly differ at *p* < 0.05

RT-qPCR was conducted to analyze the expression levels of seven stress-related genes (*TdNHX1*, *TdSOS1*, *TdSOD*, *TdCAT*, *TdGA20-ox1*, *TdNRT2.1*, and *TdGS*) in the leaves of durum wheat. Generally, the application of *Ro*EO was capable of modulating gene expression in wheat seedlings. Salt-stressed wheat seedlings treated with *Ro*EO had significantly higher transcript levels of six stress-related genes (*TdNHX1*, *TdSOS1*, *TdSOD*, *TdCAT*, *TdGA20-ox1*, and *TdGS*) than under both control and saline conditions (Fig. 5). Only in the case of the *TdNRT2* gene (related to nitrate transport) were the expression levels stable, regardless of the treatment (Fig. 5).

### Principal component analysis for stress-related parameters

**Fig. 6:**
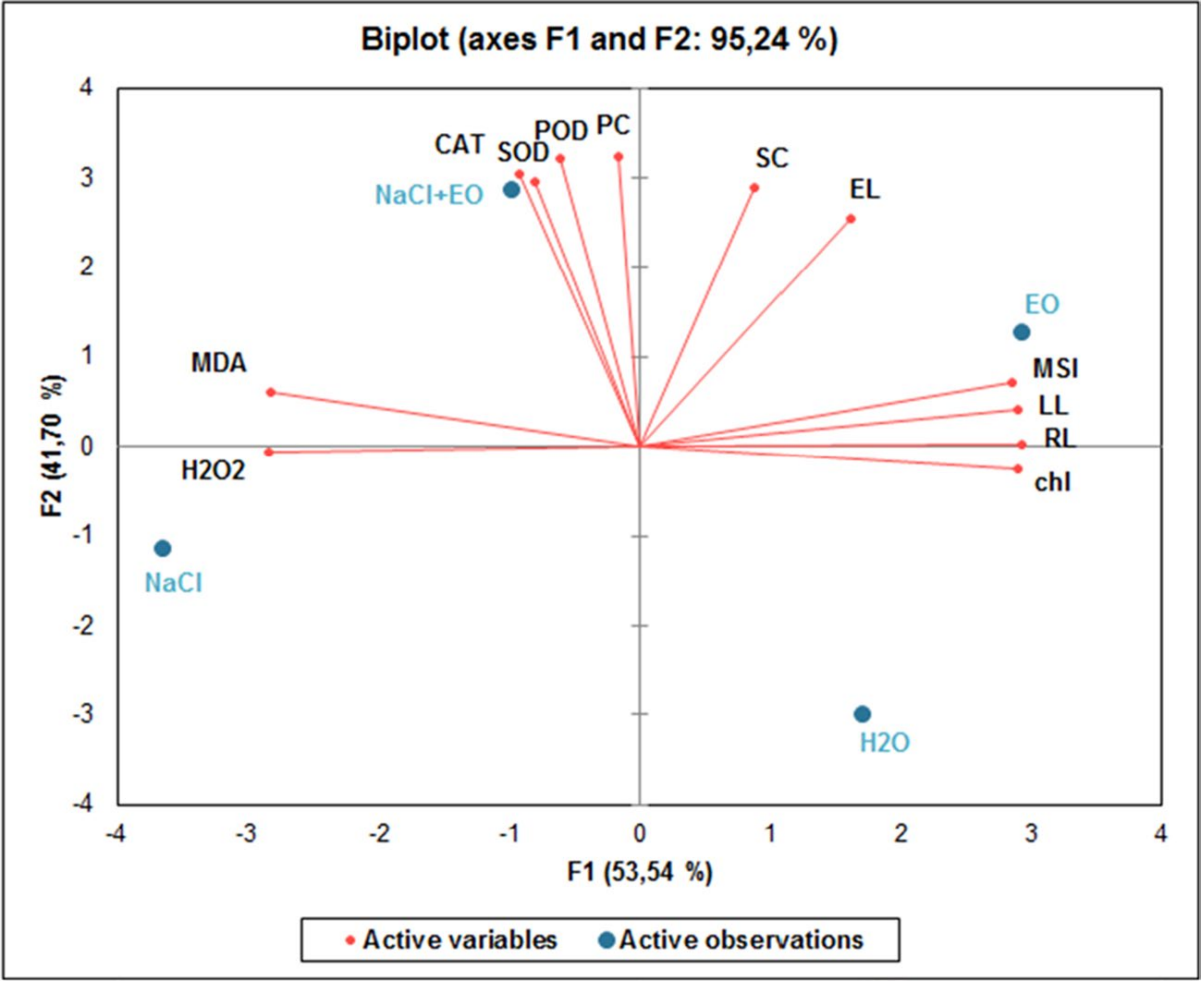
Principal component analysis (PCA). Red circles represent the parameters analyzed. Blue circles represent different growth conditions, H_2_O (control), NaCl (salt treatment at 150 mM), EO (seeds primed with *Ro*EO), and NaCl + EO (seeds primed with *Ro*EO under 150 mM NaCl). F1-F2 principal factorial plane explains 95.24% of the variation between all studied parameters and different conditions. RL, root length; LL, leaf length; chl, chlorophyll; MSI, membrane stability index; EL, electrolyte leakage; SC, sugar content; PC, proline content; H_2_O_2_, hydrogen peroxide content; MDA, malondialdehyde content; SOD, superoxide dismutase activity; CAT, catalase activity; POD, peroxidase activity

To identify possible correlations between treatments and the different evaluated parameters, principal component analysis (PCA) was carried out (Fig. 6, Table 2). Both principal factorial planes 1 and 2 have eigenvalues greater than 5 and contributed to 53.54% and 41.70% of the total variance, respectively (Table 2). The most important characters contributing to F1 were root length, leaf length, chlorophyll content, membrane stability index, malondialdehyde content, and hydrogen peroxide content (score > 9.4). All other parameters were responsible for the variance in F2. Seedlings treated with *Ro*EO were clustered with growth-related parameters on the positive side of F1. Seedlings subjected to salt stress were clustered with malondialdehyde and hydrogen peroxide content traits on the negative side of F1, while *Ro*EO-treated seedlings under salt stress conditions are clustered with salinity tolerance linked parameters, including SOD, CAT, and POD antioxidant enzyme activities and proline content almost at the center F1 and the positive side of F2. These results suggest that *Ro*EO treatments may play a role in promoting growth in wheat seedlings and enhancing their ability to deal with salinity.

**Table 2:** PCA results for the different parameters studied

	F1	F2	F3
RL	**1.000**	0.000	0.000
LL	**0.981**	0.015	0.004
chl	**0.980**	0.006	0.014
MSI	**0.953**	0.046	0.000
EL	0.304	**0.595**	0.101
SC	0.091	**0.765**	0.144
PC	0.003	**0.952**	0.044
MDA	**0.942**	0.033	0.024
H_2_O_2_	**0.950**	0.000	0.050
CAT	0.077	**0.799**	0.124
SOD	0.099	**0.850**	0.051
POD	0.044	**0.942**	0.014

Values ≥ 0.94 are presented in bold and indicate important traits for PCA. *RL* root length, *LL* leaf length, *chl* chlorophyll, *MSI* membrane stability index, *EL* electrolyte leakage, *SC* sugar content, *PC* proline content, *H*_*2*_*O*_*2*_ hydrogen peroxide content, *MDA* malondialdehyde content, *SOD* superoxide dismutase activity, *CAT* catalase activity, *POD* peroxidase activity

## Discussion

The impact of abiotic stress on agricultural productivity is manifested through diminished crop growth, which contributes to global food crises. Presently, environmentalists are focused on securing food resources by enhancing crop production in challenging environments. Augmenting the germination potential of seeds holds paramount importance as a criterion for enhancing crop competitiveness through efficient water and nutrient utilization. However, under stress conditions such as salinity, germination is inhibited by Na^+^ and Cl^−^ toxicity, which is reflected in the osmotic potential and ROS production (Yan et al. 2013; Astaneh et al. 2018). Lutts et al. (2016) suggested that subjecting seeds to transient stress agents during germination within embryos can result in stress memory, fostering more efficient adaptation to subsequent stress episodes. The use of plant extracts and aromatic oils in agriculture has been proposed as an environmentally friendly and cost-effective approach (Farooq et al. 2018; Ben-Jabeur et al. 2022). Day (2016) findings revealed that the EO extracted from safflower plants’ stem and root tissues had varying effects on the germination rates of wheat, barley, sunflower, and chickpea. In this current study, our objective was to investigate the impact of seed treatment with *Ro*EO on the germination performance, growth establishment, and responses to stress, along with the mechanisms involved in stress amelioration under salinity conditions. It appears that the applied EO influences plant growth characteristics akin to other organic compounds, serving as a biostimulant (Marschner 2011; Souri and Römheld 2009). Numerous reports highlight the biostimulant effects of phenolic compounds and plant extracts on seed germination, rooting, and shoot development. These effects are observed when treating seeds, leaves, or soil with EOs (Kisiriko et al. 2021). In our current study, we propose that the application of 5 ppm of *Ro*EO is adequate to enhance salt stress tolerance in durum wheat plants. Nevertheless, understanding its precise mode of action is a complex matter that necessitates further investigation. Binbir et al. (2019) demonstrated that the germination rate decreased, and the dry weight of the seedlings increased as a consequence of treating corn seeds with lavender EO. Ben-Jabeur et al. (2022) illustrated that employing the seed coating technique with thyme EO resulted in enhanced vegetative growth and grain yield. This improvement was associated with the regulation of ABA (abscisic acid) in plants experiencing drought stress. Hara (2020) reported that biostimulants containing EO heat tolerance enhancers (HTLEs) such as isothiocyanates and monoterpenes may be useful in reducing yield and crop quality losses due to various stresses, especially heat. Increased yields were obtained with thyme EO. Basu et al. (2016) demonstrated that elevated chlorophyll levels resulting from EO treatments support the hypothesis that certain mechanisms are triggered to mitigate damage caused by drought stress. Consistent with these findings, our results indicate that *Ro*EO alleviated the adverse effects of salt stress on photosynthetic efficiency, as evidenced by an increase in the chlorophyll fluorescence (*Fv/Fm*) value. The substantial increases in soluble sugar contents observed in *Ro*EO-treated wheat seedlings under salt stress may be attributed to the potential role of *Ro*EO in maintaining photosynthetic efficiency. The application of thyme EO was found to minimize chlorophyll damage and limit the accumulation of anthocyanins at the onset of severe stress. These observations suggest that thyme EO plays a protective role in preserving photosynthetic capacity, preventing photo-oxidation damage, and impeding stress-induced leaf senescence (Hsiao et al. 1984), which is related to the potential of thyme EO to enhance the peroxidase-mediated antioxidative mechanisms (Ben-Jabeur et al. 2015). Soluble sugars act as osmoregulators that tend to increase in plants under salt stress and are able to reduce membrane permeability and affect osmotic pressure (Chaves 1991; Baki et al. 2000). Proline has been reported to accumulate in many plant species under a wide range of environmental stress conditions (Ashraf and Harris 2004; Claussen 2005; Xiong et al. 2014). Thus, proline is known to play several roles in plants, especially in osmotic regulation and ROS removal (Szabados and Savouré 2010). In the present study, at 150 mM NaCl, *Ro*EO treatment increased proline and leaf soluble carbohydrate contents compared to the untreated control plants. Consequently, the results indicated that *Ro*EO could accumulate osmoregulatory substances to regulate the cell osmotic balance.

Salt stress often leads to excessive accumulation of ROS and causes oxidative damage to proteins, lipids, and nucleic acids. Excessive amounts of ROS in plant cells lead to lipid peroxidation and simultaneously increase the amounts of H_2_O_2_ and MDA. In this study, H_2_O_2_ and MDA accumulation increased significantly in the leaves of durum wheat under salt stress conditions but the exogenous application of *Ro*EO reduced this increasing trend. This suggests that *Ro*EO probably acts to protect the integrity of the cell membrane by minimizing oxidative stress, leading to lipid peroxidation. According to Bailly (2019), low levels of reactive oxygen species (ROS) positively impact germination, whereas high ROS levels can lead to oxidative damage, inhibiting seed germination. The heightened germination observed with seed priming using EO is attributed to the mitigation of ROS accumulation, maintaining a low level conducive to germination (Hussain et al. 2019). Antioxidant defenses, including the activation of SOD, CAT, and POD, often play an important role in preventing damage to plant cell membrane systems due to ROS accumulation (Jaleel et al. 2009; Khan et al. 2019; Li et al. 2020). In the present study, we reported that the activities of SOD, POD, and CAT increased in the leaves of durum wheat plants in response to salinity. These enzymes were able to scavenge free radicals and ROS, which led to effective protection of the plasma membrane and eventually reduced accumulation of lipid peroxidation products in the membrane. The activation of these enzymes was further enhanced when *Ro*EO was applied. Therefore, these results demonstrated that *Ro*EO could reduce the accumulation of ROS and prevent the deterioration of membrane structure and function in durum wheat cells in response to salt stress. A similar effect of EO application was revealed in avocado fruit. Exposure to thyme EO increased the activities of antioxidant enzymes (SOD, POD, and CAT) in comparison with untreated control fruit (Sellamuthu et al. 2013). Furthermore, Ben-Jabeur et al. (2015) validated the ability of thyme EO to safeguard tomato seedlings through the accumulation of peroxidases, which serve as the first line of defense against ROS. Additionally, EOs from *Allium sativum* and *R. officinalis* were identified as preservatives for quality parameters in treated strawberry fruits against *Colletotrichum nymphaeae*. This preservation was attributed to the stimulation of peroxidase activity and the synthesis of phenolic compounds (Hosseini et al. 2020).

As genes from NHX family encode tonoplast Na^+^/H^+^ antiporters responsible for Na^+^ sequestration in vacuoles, the enhanced expression of *TdNHX1* gene detected in our study may indicate that similar mechanisms of salinity tolerance were probably activated by *Ro*EO treatment. This assumption is supported by the elevated accumulation of osmolytes (proline and soluble sugars) to align osmolarity between vacuole and the cytoplasm. On the other hand, the SOS1 protein, localized in the plasma membrane, which partially mediates Na^+^ efflux from the cell expression of the *TdSOS1* gene encoding this antiporter, was also elevated in *Ro*EO-treated wheat, suggesting that removal of excess Na^+^ was facilitated by *Ro*EO application. *Ro*EO clearly promotes both mechanisms of Na^+^ neutralization in durum wheat. The biostimulatory activity of *Ro*EOs was also manifested in boosted antioxidant activity. A greater accumulation of *TdSOD* and *TdCAT* transcripts occurred in parallel with the enhanced activity of the enzymes. Our results are in line with recent findings highlighting the crucial role of the genes encoding antioxidant enzymes in plant resilience in challenging environments. Another gene upregulated by *Ro*EO treatment was *TdGA20-ox1*, encoding the enzyme of gibberellin (GA) biosynthetic pathway, which is responsible for the synthesis of active GA forms (Li et al. 2020). Increased expression of the gene was likely associated with higher accumulation of active GA, which facilitated the growth of the seedlings. A similar tolerance mechanism was reported for other monocotyledon species, such as rice (Li et al. 2020), grown under saline or saline-alkaline conditions.

The expression pattern of the *TdNRT2.1* gene encoding nitrate transporter was not affected by either salinity or *Ro*EO application. This may indicate that applied conditions did not interfere with nitrogen assimilation in durum wheat, unlike in salinity-stressed or maize (Ertani et al. 2013). On the other hand, we observed enhanced expression of the *TdGS* gene involved in nitrogen metabolism, particularly amino acid biosynthesis, including proline. Higher expression of this gene may be interrelated with intensified synthesis of free amino acids as osmoprotectants under *Ro*EO application.

## Conclusions

In conclusion, our study demonstrated the positive effects of *Ro*EO during the early stages of seedling development as well as on salinity tolerance in the durum wheat cultivar “Mahmoudi.” At an optimal dose (5 ppm), *Ro*EO acted as a biostimulant, increasing the germination rate and biomass accretion and ameliorating physiological performance under saline conditions. Our comprehensive biochemical and molecular analyses revealed that *Ro*EO boosted the main defense mechanisms towards salinity stress: enhancement of photosynthetic efficiency, synthesis of osmoprotectants for osmotic adjustment, transcriptional modulation of salt stress-related genes, and ROS scavenging. Overall, the application of *Ro*EO represents a promising environmentally friendly alternative approach for enhancing wheat tolerance and preserving plant yield potential under salt stress conditions, making it a preferred choice for sustainable agricultural practices. Considering the chemical composition of *Ro*EO, future studies will be carried out to better understand the mechanism by which this EO exerts its biostimulatory effect through a targeted study on the main compounds.

## Supplementary information

Below is the link to the electronic supplementary material.

Supplementary file1 (DOCX 16 KB)

## Data Availability

All generated data are included in this article.
