# Supplementary material for: Rosmarinus officinalis L. essential oil enhances salt stress tolerance of durum wheat seedlings through ROS detoxification and stimulation of antioxidant defense
Source: Protoplasma. 2024 Jun 28;261(6):1207–20. doi: 10.1007/s00709-024-01965-8 (PMC11511768; doi:10.1007/s00709-024-01965-8)
Supplement: Supplementary file 1 — Supplementary file1 (DOCX 16 KB) [file 709_2024_1965_MOESM1_ESM.docx]

**SUPPLEMENTARY MATERIAL**

***Rosmarinus officinalis* L. essential oil enhances salt stress tolerance of durum wheat seedlings through ROS detoxification and stimulation of antioxidant defense**

Rania Ben Saad^1^, Walid Ben Romdhane^2^, [Alina Wiszniewska](https://sciprofiles.com/profile/617405?utm_source=mdpi.com&utm_medium=website&utm_campaign=avatar_name)^3^, Narjes Baazaoui^4^, Mohamed Taieb Bouteraa^1,5^, Yosra Chouaibi^1^, Mohammad Y. Alfaifi^6^, Miroslava Kačániová ^7,8^**,** Natália Čmiková ^7^, Anis Ben Hsouna^1,9^, Stefania Garzoli^10,*^

**Table S1.** Sequences of primers used for real-time analysis.

| **Primers** |  | | **Sequences** | **Accession no.** |
| --- | --- | --- | --- | --- |
| *qRT-NHX1-F* | *Sodium/hydrogen exchanger 1* | 5'-GCAGCTGATATCTCAAGCCA-3' | | [AY296910.1](https://www.ncbi.nlm.nih.gov/nuccore/AY296910.1) |
| *qRT-NHX1-R* |  | 5'- ACTGAAGGTTTGTTTGGGGG-3' | |  |
| *qRT-SOS1-F* | *Salt overly sensitive 1* | 5’- AGAGTTTCCGGAGATCCAGC-3’ | | [EU552490.1](https://www.ncbi.nlm.nih.gov/nuccore/EU552490.1) |
| *qRT-SOS1-R* |  | 5’- TGCTGCCATACATGCTGACT-3’ | |  |
| *qRT-CAT-F* | *Catalase* | 5’-CGAGAAGATGGTGATCGAGA-3’ | | [KP696753.1](https://www.ncbi.nlm.nih.gov/nuccore/KP696753.1) |
| *qRT-CAT-R* |  | 5’-TGTTGATGAATCGCTCTTGC-3’ | |  |
| *qRT-SOD-F* | *Superoxide dismutase* | 5'-GCCATTGATGAGGATTTTGG-3' | | [KP696754.1](https://www.ncbi.nlm.nih.gov/nuccore/KP696754.1) |
| *qRT-SOD-R* |  | 5'-CAAAGCTAGCCACACCCATC-3' | |  |
| *qCDC-F* | *Cell division control protein* | 5’-GCCTGGTAGTCGCAGGAGGAT-3’ | | Ta54227 |
| *qCDC-R* |  | 5’-ATGTCTGGCCTGTTGGTAGC-3’ | |  |
| *qGA20ox1-F* | *Gibberellin20 oxidase 1* | 5’-CGCCTACCCGGACTTCAC-3’ | | XM_037588783 |
| *qGA20ox1-R* |  | 5’-TAAGTAAGTCATGTCCTGGCGG-3’ | |  |
| *qNRT2.1-F* | *High-affinity nitrate transporter 2.1* | 5’-GGAGGCTCGACTACTTCTAC-3’ | | XM_037589359 |
| *qNRT2.1-R* |  | 5-ATCATGGCCTCCTCGTCAG-3’ | |  |
| *qGS-F* | *Glutamine synthetase* | 5’- GAGACCGCCGACATCAACAC-3’ | | XM_037569872 |
| *qGS-R* |  | 5’- TCATGGAGGTGACGACGTAG-3’ | |  |

|  |  |
| --- | --- |
